# Development of Silk Fibroin Scaffolds by Using Indirect 3D-Bioprinting Technology

**DOI:** 10.3390/mi13010043

**Published:** 2021-12-28

**Authors:** Yeong-Jin Choi, Dong-Woo Cho, Hyungseok Lee

**Affiliations:** 1 Department of Advanced Biomaterials Research, Korea Institute of Materials Science (KIMS), 797, Changwon-daero, Seongsan-gu, Changwon-si 51508, Korea; jinchoi@kims.re.kr; 2 Department of Mechanical Engineering, Pohang University of Science and Technology (POSTECH), Pohang 37673, Korea; 3 Institute for Convergence Research and Education in Advanced Technology, Yonsei University, Seoul 03722, Korea; 4 Department of Mechanical and Biomedical Engineering, Kangwon National University (KNU), 1 Gangwondaehakgil, Chuncheon-si 24341, Korea; 5 Interdisciplinary Program in Biohealth-Machinery Convergence Engineering, Kangwon National University (KNU), 1 Gangwondaehakgil, Chuncheon-si 24341, Korea

**Keywords:** silk fibroin, scaffold, indirect, 3D-bioprinting, natural polymer, tunable mechanical property

## Abstract

Due to the excellent biocompatibility of natural polymers, a variety of natural polymers have been widely used as biomaterials for manufacturing tissue engineered scaffolds. Despite the excellent biological activity of natural polymers, there have been obstacles in using them on their own to prepare 3D scaffolds with sufficient mechanical strength. Although multiple 3D-bioprinting technologies have recently emerged as effective manufacturing tools for scaffold preparation, scaffold preparation using only natural polymers with tunable mechanical properties is still difficult. Herein, we introduce novel scaffold fabrication methods using the natural polymer silk fibroin via indirect 3D-bioprinting technology. The developed silk fibroin scaffolds showed biocompatibility and tunable mechanical strength by changing the concentration of the silk fibroin. Furthermore, controlling the flexibility of the silk fibroin scaffolds was made possible by changing the solvent for the silk fibroin solution used to fabricate the scaffold. Consequently, silk fibroin scaffolds fabricated via our method can be considered for various applications in the bioengineering of either soft or musculoskeletal tissues.

## 1. Introduction

Conventionally, autologous or allogeneic tissue is transplanted to regenerate and re-store damaged tissue, which can be accompanied by various side effects such as donor morbidity and disease transmission [1]. Scaffolds widely used in tissue engineering are used as support structures for tissue growth. For effective tissue regeneration, scaffolds that simulate the structure, mechanical strength, and biochemical properties of the extra-cellular matrix (ECM) should be fabricated [2,3,4]. A variety of techniques have been used to fabricate scaffolds, such as electrospinning, freeze-drying, decellularization, and micro-patterning [5,6]. However, these technologies have shape and structural control limitations that make it difficult to simulate the complex structure of the ECM.

3D-bioprinting, a recently emerged technology in the field of tissue engineering, can fabricate complex-shaped structures by precisely printing various biomaterials, biomolecules, and cells at the desired location through spatial control [7,8]. Three dimensional-bioprinting technology uses computer-aided design (CAD) and computer-aided manufacturing (CAM) to process medical images from sources such as computed tomography (CT) to accurately determine the site of the patient’s injury [9,10,11]. Therefore, 3D-bioprinting is an advantageous technique for replicating the architecture of the complex ECM structure.

Silk fibroin (SF), a natural fibrous protein from *Bombyx mori*, has been identified as a potential biodegradable material for tissue engineering due to its biocompatibility and excellent mechanical properties [12]. SF can be made into films, hydrogels, electrospun membranes, and porous sponges and has been widely applied as scaffolds in tissue engineering [13,14,15]. SF is used as a bioink for bioprinting through shear-induced beta crystallization, enzymatic crosslinking, chemical modification, and incorporating other materials to enhance its functionality [16,17,18]. Since SF has low deposition ability for extrusion-based bioprinting due to its low viscosity, it can be used as a bioink by increasing its concentration and mixing it with gelatin or glycerol. In the light-assisted bioprinting system, SF structures of various shapes have been fabricated by making SF photocurable through methacrylation [17]. However, the mechanical strength of SF scaffolds fabricated via these approaches is only a few kPa, thereby making it difficult to apply them to tissue regeneration [18]. When a scaffold is implanted in the defect site, it is surrounded by the mechanical stress induced by the tissue environment. The low mechanical strength of the scaffold can affect the performance of the delivered cells at the defect site and inhibit their growth within the tissue [19]. Therefore, it is important to fabricate a scaffold with mechanical strength suitable for the tissue surrounding the defect site. Nevertheless, it is difficult to fabricate complex structures while maintaining the high mechanical strength of SF. Although various attempts to use chemical crosslinking with and chemical modification of SF have been made, it is difficult to simultaneously meet the requirements for a complex structure and high mechanical strength through 3D-bioprinting.

In our previous work, we developed an indirect 3D-printing technique that enables fabricating constructs with complex geometries [20,21]. Through this technology, a structure fabricated with light-assisted 3D-printing technology can be used as a mold into which various biomaterials can be injected and cured, after which the mold can be removed. This mold-casting process-based indirect printing technology can produce complex shapes without considering the physical properties of the biomaterials, such as viscosity. In addition, indirect 3D-printing technology can produce various shapes using both natural and synthetic polymers. Indirect 3D-printing using natural polymers such as gelatin has been used to produce a nasal implant with an octahedral internal structure [19].

In the present study, SF scaffolds were fabricated using indirect 3D-printing technology. SF was prepared based on two solvents (aqueous SF and hexafluoroisopropanol (HFIP)-based SF) to generate SF scaffolds with various properties. A sacrificial mold containing a porous scaffold structure was fabricated through indirect 3D-printing using projection-based microstereolithography (pMSTL) an in-house-developed 3D-bioprinting system. SF at different concentrations (10% and 20% for aqueous; 10%, 20%, and 30% for HFIP-based) were injected into sacrificial molds, which were then removed after curing and inducing crystallization of the SF solution. Visual observation and scanning electron microscopy (SEM) confirmed that the surface properties of the SF scaffolds of both solvent types differed in terms of color and roughness. Mechanical strength measurements confirmed that the compressive modulus value increased as the concentration of SF was increased, and it was observed that the HFIP-based SF scaffold had significantly higher mechanical strength than the aqueous one. Human bone marrow-derived mesenchymal stem cells (hBMMSCs)s were seeded onto each scaffold to check for changes in their cellular characteristics; it was confirmed that cell attachment was lower as the concentration of SF was increased.

SF scaffolds with tunable mechanical strength and a complex porous architecture were fabricated through aqueous and HFIP-based SF using indirect 3D-printing technology. The tunable mechanical strength of these SF scaffolds makes them practicable for repairing defect sites involving tissue regeneration.

## 2. Material and Methods

### 2.1. Preparation of the SF Solutions

The SF solution was prepared as described previously [16]. Briefly, silk cocoons were cut into small pieces and boiled for 30 min in 0.02 M sodium carbonate (Na_2_CO_3_) to remove silk sericin. The SF fibers were rinsed three times with deionized water (DW) for 20 min and then dried. They were then dissolved in a 9.3 M lithium bromide (LiBr) solution at 60 °C for 4 h to make a 20% *w*/*v* solution. This was then dialyzed against DW using 3.5 K molecular-weight cutoff (MWCO) dialysis tubing (Thermo Fisher Scientific; Waltham, MA, USA) at room temperature for 48 h to remove the LiBr. The SF solution was centrifuged at 4000 rpm and filtered through a cell strainer to remove undissolved contaminants. The concentration of the aqueous SF solution was 8% *w*/*v*.

**Figure 1:**
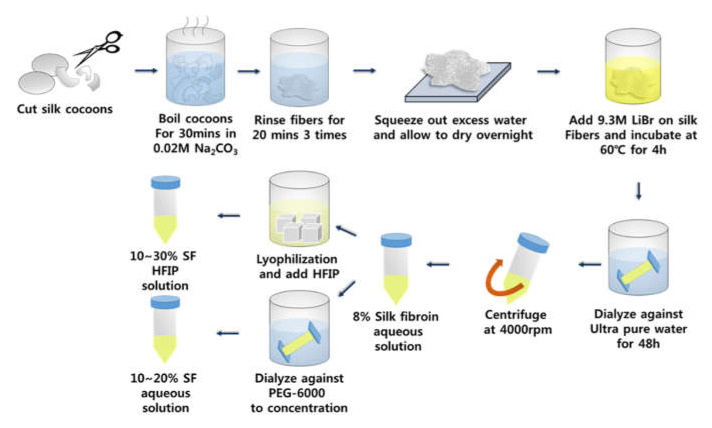
A schematic diagram of SF solution preparation.

For preparing the HFIP-based SF solutions, aqueous SF solution was first lyophilized, after which the lyophilized SF was dissolved in HFIP to produce 10%, 20%, and 30% SF solutions. The 10% and 20 wt% aqueous SF solutions were prepared by dialyzing against 10 wt% polyethylene glycol (6000 MW, Sigma-Aldrich, Burlington, MA, USA) solution for 12–24 h (Figure 1).

### 2.2. Preparation of the Sacrificial Scaffold Mold

**Figure 2:**
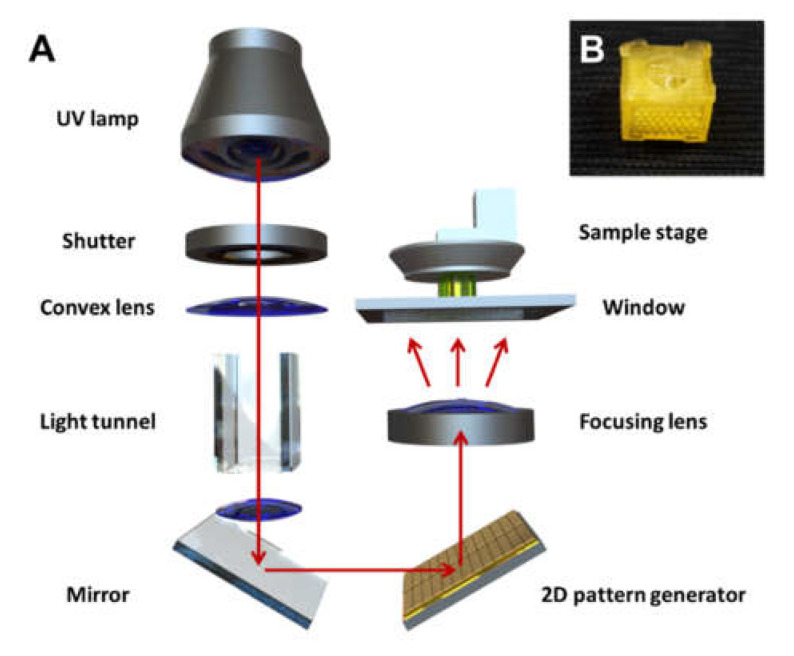
(**A**) A schematic diagram of the indirect 3D-printing system for sacrificial scaffold mold preparation and (**B**) a 3D-printed sacrificial mold.

Before SF scaffold fabrication, sacrificial scaffold molds consisting of the alkali-soluble photopolymer resin were printed using projection-based microstereolithography (pMSTL) technology [20]. Sacrificial scaffold mold images data were generated perpendicularly onto the resin by using a light and a projector (Figure 2A). Solidified photopolymer layers with 2D patterns were successively stacked layer-by-layer to create a sacrificial 3D-printed mold for the scaffold (Figure 2B).

### 2.3. Preparation of the Injectable Silk Biomaterials and Scaffold Fabrication

**Figure 3:**
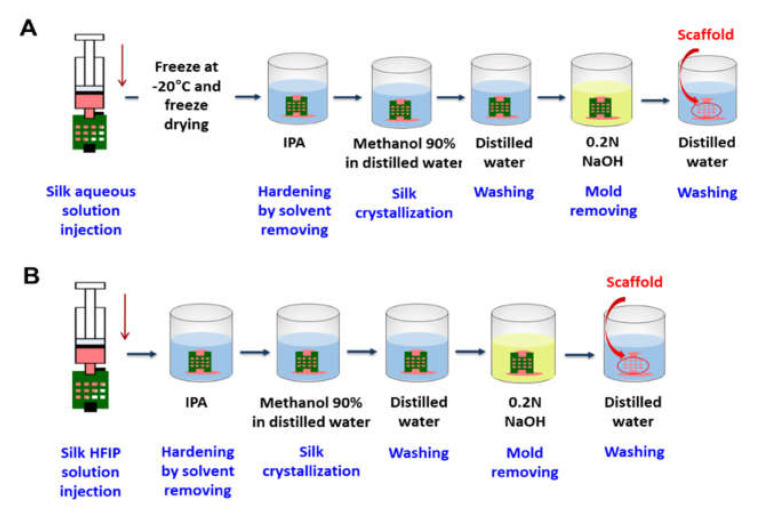
Schematic diagrams of the sacrificial molding process and SF scaffold fabrication depending on the solvent: (**A**) water and (**B**) HFIP.

To develop SF scaffolds 4 × 4 × 3 mm^3^ in size, we used two different SF solutions dissolved in either DW or HFIP to evaluate whether the properties of the scaffold depended on the solvent type. The sacrificial molding process for the aqueous SF scaffold was as follows: (1) Inject SF aqueous solution into the prepared mold. (2) Freeze and lyophilize the SF to maintain the structure of the scaffold. (3) Harden by removing the solvent through isopropyl alcohol (IPA) treatment. (4) Treat with methanol to induce silk crystallization. (5) Wash with DW. (6) Treat with sodium hydroxide (NaOH) to remove the mold. (7) Wash with DW (Figure 3A).

The HFIP-based SF scaffold process was similar but did not require step (2) due to it being able to maintain the excellent structure of the sacrificial mold. Salt-leached scaffolds were also prepared for comparison (Figure 3B).

### 2.4. SEM Analysis

SEM (SU-6600, Hitachi, Tokyo, Japan) was conducted to examine the outer and inner structures (including pore size and uniformity) of the SF scaffolds. Printed channels were dried under vacuum at room temperature. The SF scaffolds were dried and lyophilized (FDU-8603, OPERON, Gimpo, Korea), after which they were sputter-coated with platinum (Ion Sputter E-1045, Hitachi, Tokyo, Japan). The SEM system was operated at 15 kV.

### 2.5. In Vitro Cell Proliferation and Viability Test

The SF scaffolds were immersed in 70% ethanol and placed under UV light for 1 h to sterilize them. hBMMSCs (Promocell, Heidelberg, Germany) were cultured in Dulbecco’s modified Eagle’s medium (DMEM) with high glucose supplemented with 10% (*v/v*) fetal bovine serum (FBS) and 1% penicillin/streptomycin. Afterward, hBMMSCs from passage 5 were seeded onto each scaffold (4 × 4 × 3 mm^3^) at a density of 1 × 10^5^ cells/mL, followed by incubation at 37 °C for 1 h in a humid atmosphere with 5% CO_2_. For efficient cell seeding, 20 μL of hBMMSC suspension (which does not overflow while wetting the entire scaffold) was seeded on the scaffold. Culture medium was supplied to the scaffolds every 15 min to prevent them from drying out. After 30 min of cell seeding, the scaffolds were inverted to allow the cells to evenly distribute throughout them. After cell seeding, the culture medium was changed every 2 days.

Cell Counting Kit-8 (CCK-8; Dojindo Laboratory, Kumamoto, Japan) was used to evaluate cell attachment and proliferation. On days 1, 4, and 7, the medium in the well plate was exchanged with fresh medium containing 10% CCK-8 solution. Subsequently, the well plate was incubated at 37 °C in a humidified 5% CO_2_ atmosphere for 4 h to activate the CCK-8. Absorbance by the medium at a wavelength of 450 nm was measured by using a microplate reader (Wallac Victor 1420, Perkin Elmer Life Sciences, Waltham, MA, USA).

Cell viability on the scaffolds was evaluated at days 1 and 7 using a Live and Dead viability kit (LIVE/DEAD^®^ Viability/Cytotoxicity Kit, Invitrogen, Carlsbad, CA, USA). The scaffolds were washed with phosphate buffer saline (PBS) and incubated in PBS containing calcein-AM (stains live cells) and ethidium homodimer (stains dead cells) at 37 °C for 30 min. The labeled cells were observed by using confocal laser scanning microscopy (LSM800 w/Airyscan, Carl Zeiss, Oberkochen, Germany).

### 2.6. Mechanical Compression Testing

The compressive modulus values of the scaffolds were obtained by using an Instron 3340 mechanical testing system (Instron, Norwood, MA, USA). A scaffold was positioned at the center of the mechanical testing system, and then compression testing was conducted at a crosshead velocity of 1 mm/min. The compressive modulus values of the scaffolds were calculated from the linear slope of their stress-strain curve.

### 2.7. Statistical Analysis

All variables are expressed as the mean ± standard deviation (SD). Differences between the mean values of the groups were evaluated using Student’s *t*-tests. A *p*-value of <0.05 was considered to indicate significance.

## 3. Results and Discussion

### 3.1. Fabrication of SF Scaffold

**Figure 4:**
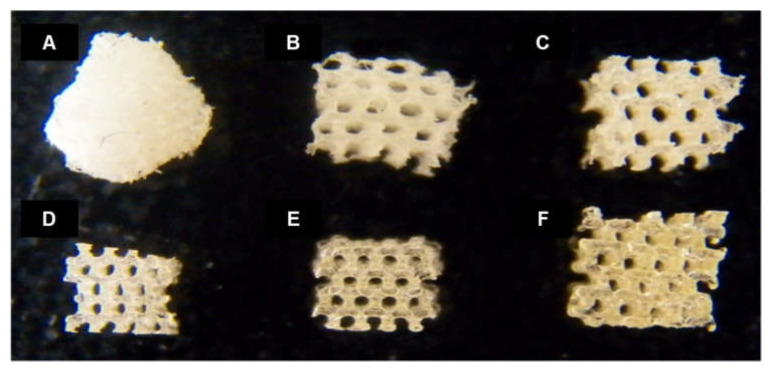
Images of (**A**) salt-leached, (**B**) 10% aqueous, (**C**) 20% aqueous, (**D**) 10% HFIP-based, (**E**) 20% HFIP-based, and (**F**) 30% HFIP-based SF scaffolds. Controlled pore shape and size were observed in all of the SF scaffolds manufactured through indirect 3D printing. The aqueous-based SF scaffold was white while the HFIP-based SF scaffold was a transparent yellowish color.

The shape fidelity and structures of the fabricated aqueous and HFIP-based SF scaffolds were evaluated and compared with a conventionally produced salt-leached SF scaffold as a comparison group (Figure 4). Even though all of the scaffolds were designed to have the same outer shape structure, that of the salt-leached SF scaffold collapsed due to its poor maintenance ability. In contrast, the SF scaffolds fabricated via the 3D-printed mold with an inner pore structure showed well-maintained outer scaffold structures.

**Figure 5:**
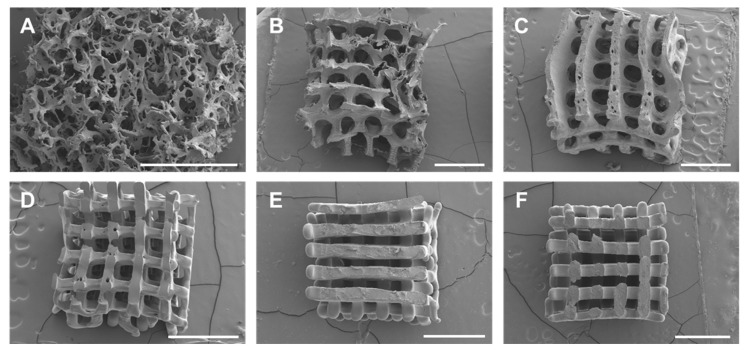
SEM images of (**A**) salt-leached, (**B**) 10% aqueous, (**C**) 20% aqueous, (**D**) 10% HFIP-based, (**E**) 20% HFIP-based, and (**F**) 30% HFIP-based SF scaffolds. The pore size, shape, and geometry of the aqueous and HFIP-based SF scaffolds fabricated via 3D-printed molds were relatively well controlled whereas the salt-leached scaffolds exhibited non-uniform pore size and shape (scale bar: 1 mm).

Conventional methods of scaffold fabrication are known to produce non-uniform pore sizes and shapes that can lead to insufficient nutrient/oxygen supply and irregular mechanical properties. As shown in the SEM images in Figure 5, the salt-leached scaffold had non-uniform pore size and shape whereas the aqueous and HFIP-based SF scaffolds prepared via the 3D-printed mold showed relatively well-controlled pore size, shape, and location. As the concentration of the SF solution was increased, more uniform pore size and shape were obtained in both the aqueous and HFIP-based SF scaffolds. Consequently, the SF scaffolds fabricated by using a 3D-printed sacrificial mold will produce a better nutrient/oxygen supply and more regular mechanical properties than via the conventional method.

Interestingly, the results confirm that the HFIP-based SF scaffolds had smoother surfaces than the aqueous SF scaffold. In addition, the aqueous SF scaffold was observed to have a microporous surface, which could be due to the extra freeze-drying step prior to IPA treatment. On the other hand, in the HFIP-based SF scaffold, there is no microporous structure on the surface of the scaffold because the HFIP was slowly removed by IPA, thereby allowing the SF molecules to come close to each other. This property can also affect biodegradation. It has been reported that an aqueous SF scaffold with a microporous structure has a faster biodegradation rate than the HFIP-based SF scaffold with a relatively smoother surface [22].

When using an indirect mold scaffold fabrication method, the concentration of the injected biomaterial solution is very important for securing high shape fidelity [21,23]. Therefore, we prepared the maximum concentration of SF in each solvent that could be used to fabricate the scaffolds. The aqueous and HFIP-based SF scaffolds could be fabricated with SF solution concentrations of up to 20% and 30%, respectively. Since the aqueous SF solutions were prepared by dialyzing against 10 wt% polyethylene glycol, dialysis for preparing a 30% aqueous SF solution was carried out for a longer time than the 10% and 20% ones. In this process, β-sheet formation of the aqueous SF solution can occur, resulting in gelation. On the other hand, HFIP, an organic solvent, has excellent SF solubility that made it possible to fabricate a scaffold with an SF concentration of 30%.

### 3.2. Biocompatibility of the SF Scaffolds

**Figure 6:**
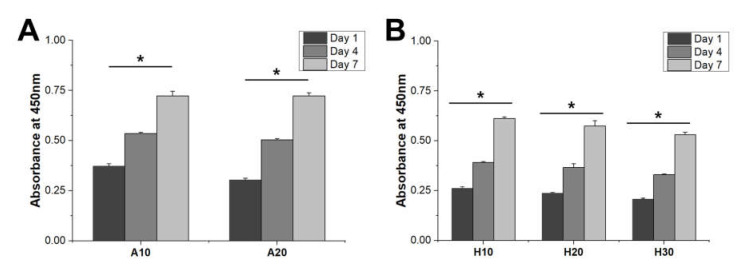
Biocompatibility of the SF scaffold. Initial cell attachment on the (**A**) aqueous and (**B**) HFIP-based SF scaffolds. All scaffolds revealed similar proliferation rates. Cell proliferation occurred in all groups at similar rates. Cell adhesion decreased in all groups with increasing SF concentration. Higher cell adhesion was observed in the aqueous-based SF scaffolds compared to the HFIP-based SF scaffolds (* *p* < 0.005).

Cell proliferation behavior was observed to confirm the biocompatibility of SF scaffolds. hBMMSCs were seeded onto the SF scaffolds and cultured. The scaffolds showed similar cell proliferation rates, confirming that they were biocompatible and could provide a suitable proliferation environment for the cells. However, the cell adhesion capacities of the SF scaffolds were different. On day 1, cell adhesion was confirmed that cell attachment decreased as the concentration of SF in the aqueous and HFIP-based SF scaffolds increased (Figure 6A,B).

Two dehydration steps were performed in the fabrication of the SF scaffolds: treatment with IPA and methanol. When SF is treated with these hydrophilic alcohols, dehydration of the hydrophobic domain in the SF occurs, resulting in the formation of β-sheet structures [24]. This phenomenon makes SF insoluble in water, which could have increased the mechanical strength of the SF scaffold, as well as controlling the biodegradation rate and structural stability [25]. Because IPA is less hydrophilic than methanol and less miscible with water, low dehydration and β-sheet formation can occur when used to treat the SF scaffolds. Thus, methanol treatment immediately after IPA treatment can increase the β-sheet formation of SF scaffolds to produce a mechanically stable structure.

Increasing the SF concentration can increase the hydrophobicity of an SF scaffold due to the increase in the hydrophobic domain. Therefore, cell adhesion was reduced as the concentration of SF in the scaffold increased.

In addition, a higher level of cell adhesion was observed on the aqueous SF scaffolds compared to the HFIP-based SF scaffolds. We consider that the aqueous SF scaffold contained better interconnected pores and a rougher surface than the HFIP-based SF scaffold, which improved initial cell attachment.

**Figure 7:**
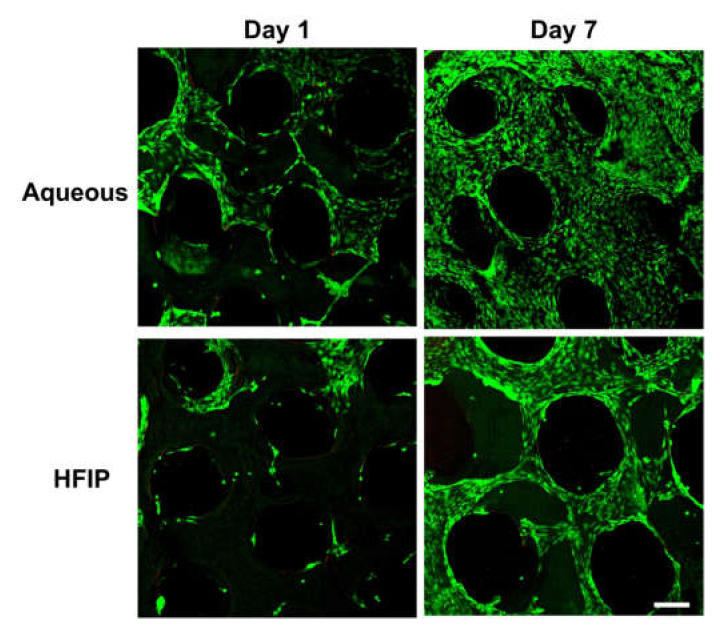
Cell viability on Aqueous-based and HFIP-based SF scaffolds. The aqueous-based SF scaffolds showed higher cell adhesion than on the HFIP-based SF scaffolds, and by day 7, cells were distributed throughout the scaffolds (scale bar: 200 μm).

In addition to the initial attachment of cells, their initial viability on a scaffold is also very important for tissue regeneration. High cell viability was confirmed on both the aqueous and HFIP-based SF scaffolds on days 1 and 7 (Figure 7). Moreover, the cells were attached along the struts of the scaffolds. In particular, by day 7, cells had stretched along and covered the entire scaffold surface. As confirmed by the cell proliferation test results, the HFIP-based SF scaffolds showed lower cell adhesion than on the aqueous-based SF scaffolds on day 1 while a relatively small number of cells covered the HFIP-based scaffolds compared to the aqueous-based SF scaffolds on day 7.

### 3.3. The Mechanical Properties of the SF Scaffolds

**Figure 8:**
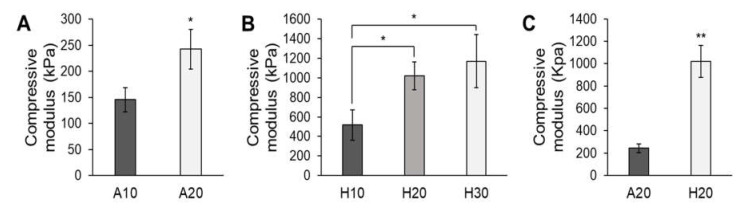
Compressive modulus values of (**A**) aqueous and (**B**) HFIP-based SF scaffolds. (**C**) Comparison of the compressive modulus values of the 20% aqueous and HFIP-based SF scaffolds. The mechanical strength of the SF scaffolds increased with increasing SF concentration. The HFIP-based SF scaffold showed higher mechanical strength than the aqueous-based SF scaffold (* *p* < 0.01, ** *p* < 0.005).

Similar to the cell adhesion results, the compressive modulus values of both the aqueous and HFIP-based SF scaffolds increased with increasing SF concentration (Figure 8A,B). The aqueous SF scaffolds were more ductile and sponge-like whereas the HFIP-based SF scaffolds were stiffer. Mechanical testing shows that the compressive modulus values of the HFIP-based SF scaffolds were significantly higher than those of the aqueous SF scaffolds (Figure 8C). Whereas the aqueous SF scaffolds had rough and porous surfaces, the HFIP-based SF scaffolds had smooth surfaces with dense struts and thus attained higher mechanical strength.

**Figure 9:**
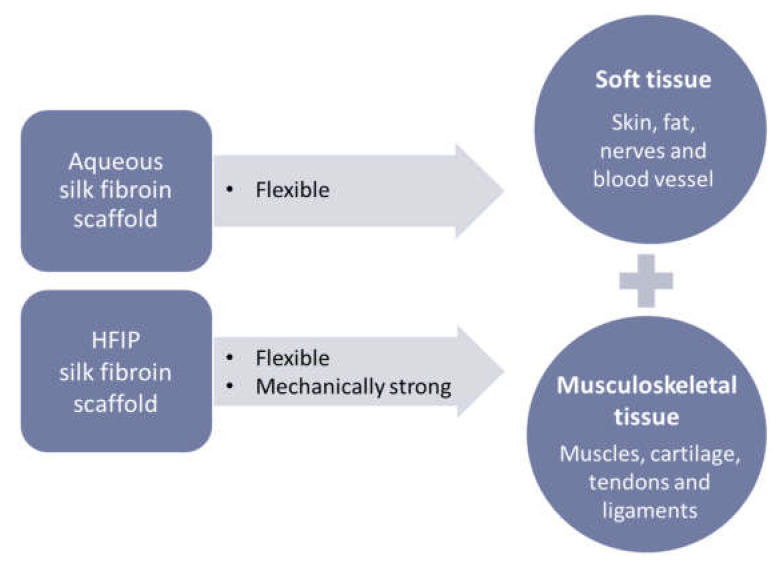
A schematic diagram of the application of SF scaffolds for tissue regeneration.

Interestingly, the SF scaffolds had different mechanical strength values depending on the SF concentration and process, which makes them suitable for use in various biological environments. Since natural human articular cartilage has a compressive modulus of approximately 1000 kPa, scaffolds with similar mechanical strength can be achieved by adjusting the concentration of HFIP-based SF [26]. In addition, the compressive modulus of human skin is around 300 kPa, which can be achieved by adjusting the concentration of the aqueous SF in the scaffold. Thus, aqueous and HFIP-based SF scaffolds with tunable mechanical strength are widely applicable to soft tissue injuries requiring flexibility and musculoskeletal tissue regeneration requiring high mechanical strength, respectively (Figure 9). In addition, through indirect 3D-printing technology, not only the shape of the SF scaffold but also the size and design of the pores can be controlled, and thus a scaffold with an ideal pore shape and size can be matched according to the biological function (e.g., 1–20 μm for cellular growth and 100–1000 μm for bone growth) [27].

## 4. Conclusions

In this study, 3D-printed porous SF scaffolds were fabricated using indirect 3D-bioprinting technology. The SF scaffolds were fabricated with 10% or 20% aqueous SF solution or with 10%, 20%, or 30% HFIP-based SF solution. The aqueous SF scaffolds had rough and porous surfaces whereas the HFIP-based SF scaffolds had rough and dense struts. In initial cell adhesion, cell attachment decreased as the concentration of SF increased. In addition, the compressive modulus values of the SF scaffolds increased as the concentration of SF was increased. SF can be applied to fabricate scaffolds with tunable mechanical strength according to concentration, solvent, and process for various tissue regeneration applications.

## Data Availability

The data presented in this study are available on request from the corresponding author.
